# Azathioprine Inhibits Hepatitis A Virus Replication In Vitro

**DOI:** 10.3390/pathogens15030249

**Published:** 2026-02-26

**Authors:** Tatsuo Kanda, Reina Sasaki-Tanaka, Hiroyuki Abe, Takeshi Yokoo, Akira Sakamaki, Kazunao Hayashi, Hiroteru Kamimura, Kenya Kamimura, Ryota Masuzaki, Hirofumi Kogure, Hiroaki Okamoto, Shuji Terai

**Affiliations:** 1 Division of Gastroenterology and Hepatology, Uonuma Institute of Community Medicine, Niigata University Medical and Dental Hospital, Uonuma Kikan Hospital, 4132 Urasa, Minamiuonuma 949-7302, Japan; 2 Department of General Medicine, Uonuma Kikan Hospital, 4132 Urasa, Minamiuonuma 949-7302, Japan; 3 Division of Gastroenterology and Hepatology, Graduate School of Medical and Dental Sciences, Niigata University, 1-757 Aasahimachi-Dori, Chuo-ku, Niigata 951-8520, Japansaka-a@med.niigata-u.ac.jp (A.S.); khayashi@med.niigata-u.ac.jp (K.H.);; 4 Division of Gastroenterology and Hepatology, Department of Medicine, Nihon University School of Medicine, 30-1 Oyaguchi Kamicho, Itabashi-ku, Tokyo 173-8610, Japan; 5 Department of General Medicine, Niigata University School of Medicine, Niigata 951-9510, Japan; 6 Division of Virology, Department of Infection and Immunity, Jichi Medical University School of Medicine, 3311-1 Yakushiji, Shimotsuke 329-0498, Japan; hokamoto@jichi.ac.jp

**Keywords:** azathioprine, hepatitis A virus, immunosuppression

## Abstract

Hepatitis A virus (HAV) infection can occasionally cause acute severe hepatitis. Patients with this disease sometimes need to undergo liver transplantation with immunosuppressants. Although rare, breakthrough HAV infections, despite vaccination, appear to be more common among immunocompromised populations. The effect of immunosuppressants on HAV replication is unclear. In this study, we examined the effects of immunosuppressants on HAV HA11-1299 genotype IIIA replication in human hepatocytes, finding that azathioprine inhibited HAV replication with a half-maximal inhibitory concentration of 0.967 μmol/L. We further examined the effect of azathioprine on the replication of HAV HM175 18f genotype IB using replication-competent or replication-incompetent subgenomic replicon in HuhT7 cells. Azathioprine had significant inhibitory effects on the HAV replication-competent subgenomic replicon compared to the replication-incompetent subgenomic replicon. The effect of azathioprine on the activity of the HAV HM175 18f genotype IB-internal ribosomal entry site (IRES) was investigated in COS7-HAV-IRES cells using a reporter assay. Azathioprine at 1 μmol/L had a significant inhibitory effect on HAV IRES activity but at 0.5 μmol/L had no inhibitory effect. Azathioprine appears to inhibit HAV replication as well as HAV translation. In conclusion, we found that azathioprine inhibits HAV replication in human hepatocytes, meaning that it may be useful for patients with a HAV infection who need to use immunosuppressants.

## 1. Introduction

Foodborne hepatitis A virus (HAV) infection and outbreaks have been associated with the consumption of foods contaminated with the feces of a person shedding HAV [1,2]. Additionally, the rate of HAV transmission through sexual contact among men has gradually been increasing [3]. HAV infection causes acute self-limited hepatitis, acute liver failure, and acute-on-chronic liver failure; it can lead to the need for a liver transplantation or even death [4]. Presently, hepatitis A outbreaks are being reported from both developed and developing countries [5,6]. Therefore, the prevention of and treatments for HAV infection are crucial.

In general, HAV infection is a vaccine-preventable disease [7,8]. A recent meta-analysis showed that, although these are rare events, hepatitis A breakthrough infections, despite vaccination, are more common among immunocompromised populations [9]. Therefore, there is a need to examine the association between HAV replication and immunosuppressants more closely.

Immunosuppressants are widely used in patients with various diseases, such as autoimmune diseases, collagen diseases, rheumatic diseases, allergic diseases, and nephrotic diseases [10,11,12]. As severe hepatitis A infection occasionally leads to acute liver failure and acute-on-chronic liver failure, liver transplantation is sometimes needed to save patients’ lives. In the case of liver transplantation, as is seen in the transplantation of other organs, the use of immunosuppressants is required to prevent rejection [13,14]. Therefore, examining the effect of immunosuppressants on HAV replication is important.

As medicine has progressed, immunosuppressants have become widely used. Among immunosuppressants, mycophenolic acid, cyclosporin A, and azathioprine are used to prevent organ transplant rejection and to treat autoimmune diseases. However, it is unknown whether these immunosuppressants have an inhibitory effect on HAV replication. Therefore, in the present study, we examine the effects that these immunosuppressants on HAV replication.

## 2. Materials and Methods

### 2.1. Cell Lines and Reagents

The human hepatoma cell line Huh7 and its derivative HuhT7 cells were kindly gifted by Prof. Ralf Bartenschlager (Universität Mainz, Mainz, Germany) and Prof. Verena Gauss-Müller (University of Lübeck, Lübeck, Germany), respectively. HuhT7 cells stably express T7 RNA polymerase in the cytoplasm [15]. African green monkey kidney cell line COS7 (JCRB 9127) was purchased from the Health Science Research Resources Bank (Osaka, Japan). COS7-HAV-internal ribosomal entry site (IRES) cells stably express the simian virus 40 (SV40) promoter plasmid pSV40-HAV-IRES, as previously reported [16].

These cells were maintained in Roswell Park Memorial Institute (RPMI) 1640 medium (FUJIFILM Wako Pure Chemical Corporation, Osaka, Japan) with 10% fetal bovine serum (FBS; Serana, Pessin, Germany) and 1% penicillin/streptomycin (FUJIFILM Wako). The cells were cultured at 37 °C with a 5% CO_2_ atmosphere in an incubator for cell cultures (MCO-50AIC-PJ, PHCbi, Tokyo, Japan).

Mycophenolic acid and azathioprine were purchased from Sigma (Saint Louis, MO, USA), and cyclosporin A was purchased from FUJIFILM Wako; these were used to compose the dimethyl sulfoxide (DMSO) solution. The DMSO solution for azathioprine was heated to 37 °C and oscillated in an ultrasonic bath to increase solubility.

### 2.2. HAV Infection

The cells were infected with HAV HA11-1299 genotype IIIA at a multiplicity of infection (MOI) of 0.01, as previously described [17]. Approximately 1 × 10^6^ cells were infected with 1 × 10^4^ copies/mL HAV RNA, and MOI was determined using previously described methods [18,19].

Briefly, Huh7 cells were seeded 24 h prior to infection at a density of 0.5 × 10^6^ cells/well in 6-well plates (AGC Techno Glass, Haibara-gun, Shizuoka, Japan). The cells were washed twice with phosphate-buffered saline (PBS; FUJIFILM Wako) and infected with the HAV HA11-1299 genotype IIIA in a serum-free medium [17]. At 4 h after infection, the cells were washed twice with PBS and incubated with fresh media.

Twenty-four hours post-infection, the media were exchanged using RPMI supplemented with 5% FBS with and without various drugs at different concentrations. Seventy-two hours post-infection, total cellular RNAs were extracted to measure HAV RNA [17].

### 2.3. RNA Extraction, cDNA Synthesis, and Quantitative PCR (qPCR) for HAV RNA

Total cellular RNAs were extracted with a QIAshredder (Qiagen GmbH, Hilden, Germany) and RNeasy Mini Kit (Qiagen), as previously described [17]. Reverse transcription was performed using PrimeScript RT reagent (Perfect Real Time, TaKaRa Bio, Kusatsu, Shiga, Japan) at 37 °C for 15 min, followed by incubation at 85 °C for 5 s. For the quantification of HAV RNA, the following primers were used: sense primer 5′-AGGCTACGGGTGAAACCTCTTAG-3′ and antisense primer 5′-GCCGCTGTTACCCTATCCAA-3′ [17]. For the quantification of β-actin mRNA, the following primers were used: sense primer 5′-CAGCCATGTACGTTGCTATCCAGG-3′ and antisense primer 5′-AGGTCCAGACGCAGGATGGCATG-3′ [17]. Quantitative PCR (qPCR) was performed using Power SYBR Green PCR Master Mix (Applied Biosystems, Thermo Fisher Scientific, Tokyo, Japan) on a 7500 fast real-time PCR system (Applied Biosystems) or a StepOnePlus real-time PCR system (Applied Biosystems) [17]. Real-time PCR assays were performed in triplicate, and the results of them were analyzed using the ddCt method [17].

### 2.4. HAV Subgenomic Replicon

HAV replication-competent subgenomic replicon pT7-18f-LUC contains an open-reading frame of firefly luciferase (Fluc) flanked by the first four amino acids of HAV polyprotein and by 12 C-terminal amino acids of VP1, followed by P2 (2A, 2B, and 2C) and P3 (3A, 3B, 3C, and 3D) domains of HAV polyprotein derived from the HAV HM175 18f genotype IB [15]. HAV replication-incompetent subgenomic replicon pT-18f-LUCmut contains a frameshift mutation in the 3D polymerase of pT7-18f-LUC. Plasmids pT-18f-LUC and pT-18f-LUCmut were kindly provided by Prof. Verena Gauss-Müller, and these constructs are well-documented in the references [15]. Approximately 0.5 × 10^6^ cells were seeded on a 6-well tissue culture plate (AGC Techno Glass) 24 h before transfection. Plasmid pT7-18f-LUC (0.2 μg) or pT7-18f-LUCmut (0.2 μg) was transfected into HuhT7 cells using the Effectene transfection reagents (Qiagen). Twenty-four hours post-transfection, the media were exchanged using RPMI supplemented with 5% FBS with and without azathioprine at different concentrations. Forty-eight hours post-transfection, cell extracts were prepared with Luciferase Cell Culture Lysis Reagent (Promega), and luciferase assays were performed using Luciferase Assay Reagents (Toyo Ink, Tokyo, Japan) on a luminescencer (JNRII-AB-2300, ATTO, Tokyo, Japan), according to the manufacturer’s instructions.

### 2.5. HAV IRES Activity

The treatment of COS7-HAV-IRES cells with various drugs at different concentrations resulted in changes in HAV IRES activities. After 24 h of treating these cells with drugs in a 6-well tissue culture plate (AGC Techno Glass), cell extracts were prepared using Luciferase Cell Culture Lysis Reagent (Promega), and luciferase assays were performed using Luciferase Assay Reagents (Toyo Ink, Tokyo, Japan) on a luminescencer (JNRII-AB-2300; ATTO, Tokyo, Japan) [16].

### 2.6. Half-Maximal Inhibitory Concentration (IC_50_)

IC_50_ is the concentration of each drug that produces 50% of the maximal inhibitory effect against HAV. We calculated IC_50_ using a previously described formula [17].

### 2.7. Cell Viability Assay

Human hepatoma cell cultures were seeded in 6-well plates (AGC Techno Glass) at a density of 0.5 × 10^6^ cells per well. After 24 h of cell seeding, drugs at various concentrations were added. After 48 h of drug addition, one-tenth of the volume of the medium was replaced with a dimethylthiazol carboxymethoxyphenyl sulfophenyl tetrazolium (MTS) reagent (Promega, Madison, WI, USA). For the evaluation of cell viability, MTS assays were performed in triplicate. After incubating for 2 h, at 37 °C and 5% CO_2_, the absorbance of each well was measured at 490 nm with an iMark Microplate Reader (Bio-Rad, Hercules, CA, USA) [17].

### 2.8. Statistical Analysis

Statistical analysis (two-tailed unpaired Student’s *t*-test) was performed using DA stats software version PAF01644 (NIFTY Corp., Tokyo, Japan). The data were presented as means ± SDs from a minimum of triplicates. *p*-values < 0.05 were considered statistically significant.

## 3. Results

### 3.1. Azathioprine Inhibits HAV Replication

**Figure 1:**
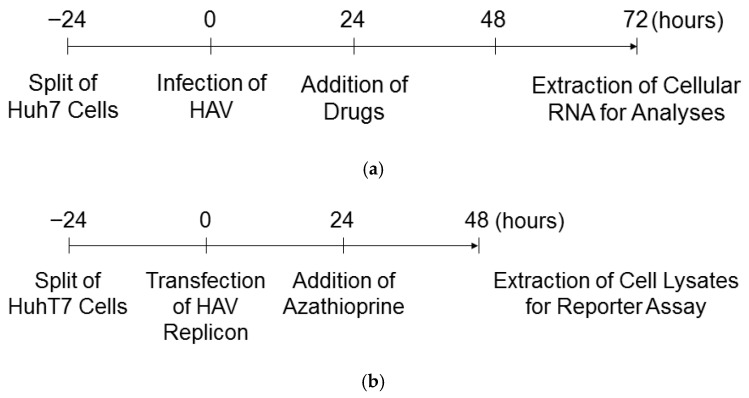

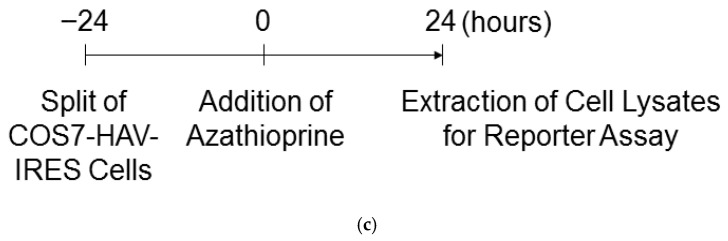
Time course experiments in this study. (**a**) Infection of Huh7 cells with HAV HA11-1299 genotype IIIA and treatment with several drugs. (**b**) Transfection of HuhT7 cells with HAV HM175/18f genotype IB subgenomic replicon and treatment with azathioprine. (**c**) COS7-HAV-IRES cells and treatment with azathioprine.

First, we examined the effects of mycophenolic acid, cyclosporin A, and azathioprine on the HAV HA11-1299 genotype IIIA replications in human hepatoma Huh7 cells (Figure 1a).

Huh7 cell monolayers in six-well culture plates were infected with HAV at an MOI of 0.01 for 4 h at 37 °C in a CO_2_ incubator. Four hours post-infection, the cells were washed twice with PBS and incubated with fresh media.

At 24 h of infection, after removing the media and washing with PBS, drug-containing media were added to the appropriate wells. After 72 h of infection, cellular RNA was extracted and real-time PCR was performed to measure HAV RNA and actin RNA.

**Figure 2:**
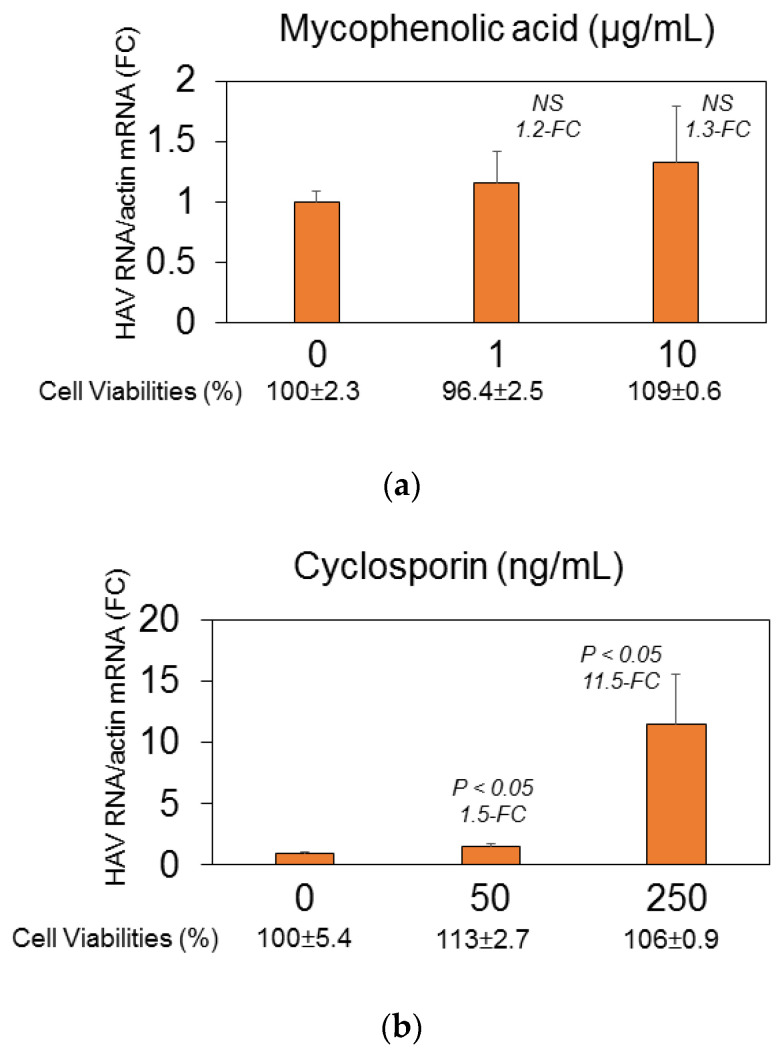

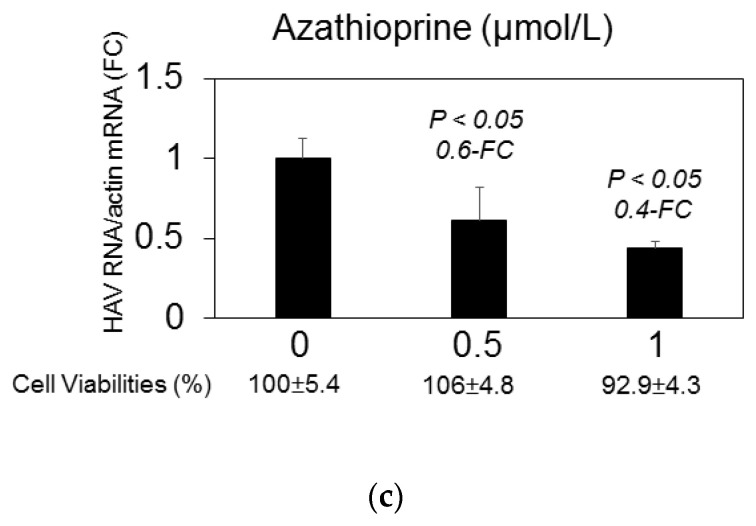
Effects of immunosuppressants on HAV HA11-1299 genotype IIIA replication in Huh7 cells. (**a**) Mycophenolic acid (molecular weight: 320.341 g/mol) and 1 μg/mL mycophenolic acid which approximately equates to 3.12 μmol/L mycophenolic acid. (**b**) Cyclosporin A (molecular weight: 1202.635 g/mol) and 1 ng/mL cyclosporin A which approximately equates to 0.832 nmol/L cyclosporin A. (**c**) Azathioprine (molecular weight: 277.26 g/mol). HAV RNA and actin mRNA were measured using RT-PCR. Data were analyzed using the ddCt method. Fold-change (FC) was compared with the untreated control. Cell viability was assessed after treatment with and without each drug for 72 h. Cell viabilities were evaluated using MTS assays. *NS*, not significant, compared with the untreated control or when *P* < 0.05 is compared with untreated control.

In Huh7, 1 μg/mL and 10 μg/mL of mycophenolic acid and 50 ng/mL and 250 ng/mL of cyclosporin A had no inhibitory effects on HAV replication without cytotoxicity [20,21]. However, azathioprine significantly inhibited HAV replication (Figure 2). The IC_50_ of azathioprine was 0.967 μmol/L. These azathioprine concentrations had no cytotoxicity to Huh7 cells for 72 h (Figure 2).

### 3.2. Azathioprine Inhibits HAV Subgenomic Replicon Replication

We analyzed the RNA replication of the HAV HM175/18f genotype IB subgenome in a DNA-based subgenomic replicon system using HuhT7 cells that stably express T7-RNA polymerase in the cytoplasm of parental cell line Huh7 [15,18]. The luciferase activities measured after the transfection of subgenomic replicon DNA are a direct measurement of RNA translation and replication of the HAV subgenomic replicon. Reporter activity due to translation and replication or translation was evaluated via the transfection of a replication-competent HAV subgenomic replicon pT-18f-LUC (wild rep) and a replication-incompetent HAV subgenomic replicon pT-18f-LUCmut (mutant rep), respectively.

**Figure 3:**
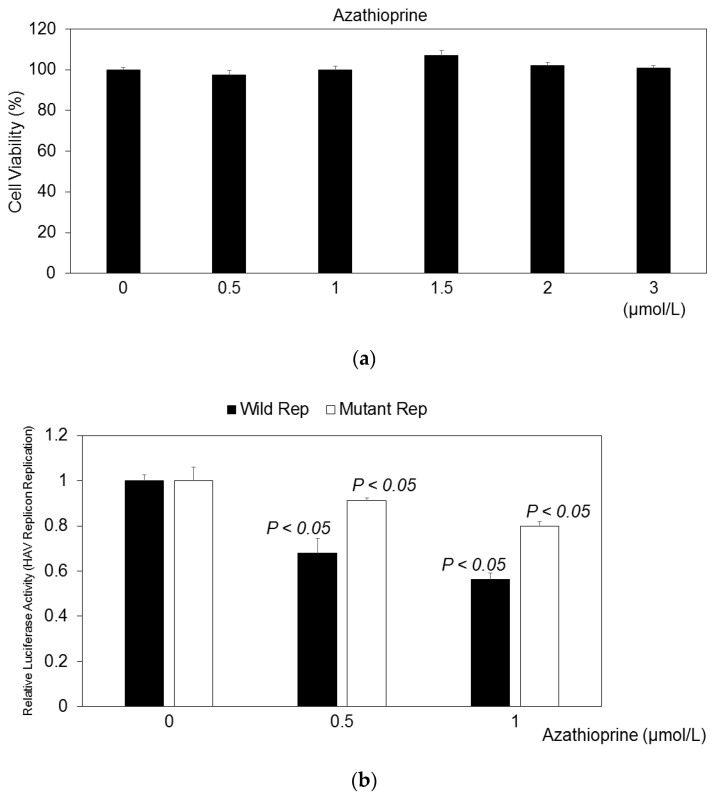
Effects of azathioprine on the HAV HM175/18f genotype IB subgenomic replicon replication in HuhT7 cells. (**a**) HuhT7 cell viability when treated with and without various concentrations of azathioprine for 48 h. Cell viability was evaluated using MTS assay. (**b**) Relative luciferase activity values of replication-competent HAV subgenomic replicon pT-18f-LUC (wild rep) and replication-incompetent HAV subgenomic replicon pT-18f-LUCmut (mutant rep) are shown as black and white bars, respectively. *P* < 0.05 is compared with each untreated control.

We examined the effects of various concentrations of azathioprine on HuhT7 cell viability for 48 h. Azathioprine at the indicated concentration exhibited no cytotoxicity against HuhT7 cells for 48 h (Figure 3a). We examined the effects of azathioprine on HAV subgenomic replicon translation and replication using this system. In HuhT7 cells, after 24 h of transfection, azathioprine was added, and after 48 h of transfection, the reporter activities were measured using the firefly luciferase assay (Figure 1b). Azathioprine at 0.5 μmol/L and 1.0 μmol/L had significant inhibitory effects on the HAV HM175/18f genotype IB replication-competent subgenomic replicon (Figure 3b; 68.0% and 56.2%, respectively, compared with the untreated control). However, azathioprine at 0.5 μmol/L and 1.0 μmol/L had lower effects on the HAV HM175/18f genotype IB replication-incompetent subgenomic replicon compared with the HAV HM175/18f genotype IB replication-competent subgenomic replicon (Figure 3b; 91.1% and 79.9%, respectively, compared with the untreated control).

### 3.3. Azathioprine Had Inhibitory Effects on HAV IRES Activity

HAV translation is a cap-independent and IRES-dependent mechanism [16]. We examined the effects of azathioprine on the activity of the HAV HM175/18f genotype IB IRES as HAV IRES is an attractive target for antivirals.

**Figure 4:**
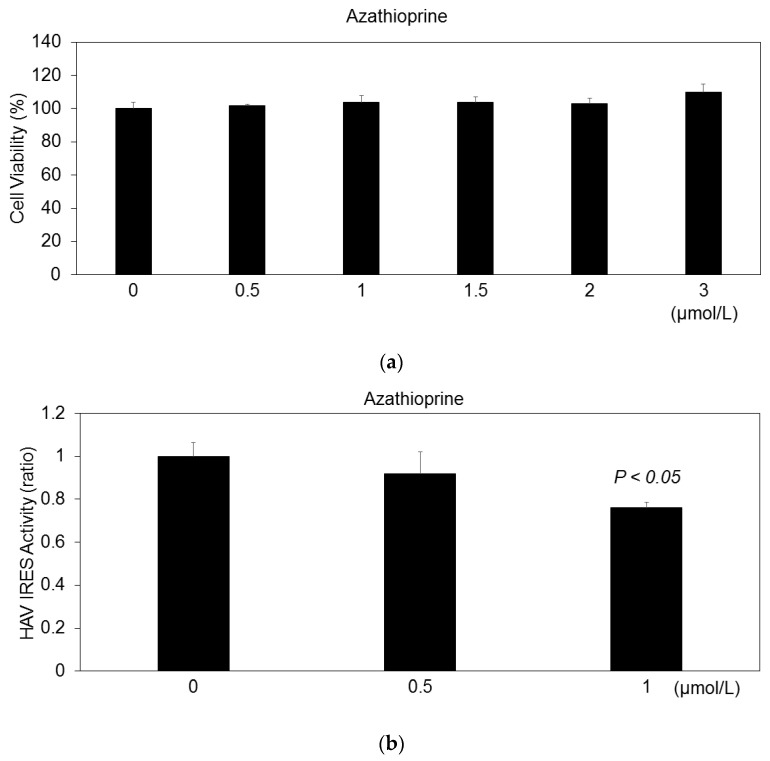
Effects of azathioprine on the HAV-internal ribosomal entry site (IRES) activity. (**a**) COS7-HAV-IRES cell viability treated with and without various concentrations of azathioprine for 48 h. Cell viability was evaluated using MTS assay. (**b**) Relative luciferase activity of HAV IRES is shown. *P* < 0.05 is compared with each untreated control. COS7-HAV-IRES cells were treated for 24 h at the indicated concentrations.

We examined the effects of various concentrations of azathioprine on COS7HAV-IRES cell viability for 48 h. Azathioprine at the indicated concentration exhibited no cytotoxicity against HuhT7 cells for 48 h (Figure 4a). We previously reported that COS7 cells stably express pSV40-HAV-IRES (COS7-HAV-IRES cells), and we successfully evaluated HAV IRES activity [16]. Approximately 0.5 × 10^6^ COS7-HAV-IRES cells were seeded on a six-well tissue culture plate 24 h before commencing the azathioprine treatment. After 24 h of treatment, HAV IRES activity was measured using the luciferase assay (Figure 1c). Azathioprine at 0.5 μmol/L did not show an inhibitory effect on the HAV IRES activity; although, at 1 μmol/L it had a significant inhibitory effect on HAV IRES activity (Figure 4b; 76.1% compared with the untreated control). These results suggest that azathioprine inhibits HAV replication rather than HAV translation (Figure 3b and Figure 4b).

## 4. Discussion

In the present study, azathioprine inhibited HAV HA11-1299 genotype IIIA replication in human hepatocytes. Using the HAV HM175/18f genotype IB subgenomic replicon and the HAV HM175/18f genotype IB IRES reporter assay, azathioprine was found to inhibit the replication step as well as the translation step in hepatocytes, thus demonstrating that azathioprine shows antiviral activity against HAV.

Azathioprine is a prodrug of 6-mercaptopurine (6-MP), which belongs to the thiopurine group of drugs that behave as purine analogs [22]. Purine nucleotides are essential organic molecules and serve as building blocks for the synthesis of DNA and RNA. In the human body, 6-MP prevents the synthesis of purine nucleotides and the growth of immune cells.

Methylated-thioinosine 5′-monophosphate (meTIMP) is an inhibitor of de novo purine biosynthesis [22]. In azathioprine metabolism, the concentrations of meTIMP are inversely correlated with the activity of inosine monophosphate dehydrogenase (IMPDH), which is an essential enzyme for the synthesis of purine [23]. Antimetabolite immunosuppressants show some similarities with previously identified antivirals, such as ribavirin, which inhibits IMPDH and shows antiviral activities [24]. It has been reported that azathioprine and its derivates show antiviral activities against various RNA and DNA viruses [22].

Azathioprine inhibits the replication of human cytomegalovirus, herpes simplex virus, and varicella zoster virus in vitro [25,26]. The doses of azathioprine we used to achieve a 50% reduction in human cytomegalovirus and for the herpes simplex virus to yield in infected human embryonic lung (HEL) cells were 0.25 μg/mL and 50 μg/mL, respectively [26]. Azathioprine and 6-MP, together with ribavirin and P13 in vitro in the culture of Hep-2 cells inoculated with recombinant human respiratory syncytial virus mGFP and wild-type human respiratory syncytial virus, showed an IC_50_ of 6.69 ± 1.41 and 3.31 ± 0.98 μmol/L for azathioprine and 6-MP, respectively. The present study shows that the IC_50_ of azathioprine in Huh7 cells infected with HAV HA11-1299 genotype IIIA is 0.967 μmol/L.

It has been reported that azathioprine metabolites, such as 6-MP, are effective for bovine viral diarrhea virus, SARS, West Nile virus, dengue virus type 2, yellow fever virus, Zika virus, and canine distemper virus. Azathioprine is used at a concentration of 1–2 mg/body weight (kg). Thiopurines, such as 6-MP, and azathioprine are converted to the inactive metabolites 6-thioxanthin (6-TX) and 6-thiouric acid (6-TUA). In renal transplant patients treated with 1.3–2.8 mg/kg azathioprine a day, the maximum plasma concentrations (Cmax) of 6-MP and 6-TUA were 73.7 ± 23.7 ng/mL and 1210 ± 785 ng/mL, respectively [27]. Genetic polymorphism nucleoside diphosphate-linked moiety X-type motif 15 (NUDT15), which belongs to the nudix hydrolase enzyme family and converts the thiopurine active metabolites, is associated with adverse reactions, in particular bone marrow toxicity and alopecia [28]. In the present study, as we did not use azathioprine metabolites, further study may be needed to determine the safe dose of azathioprine and its derivates.

Several antirheumatic drugs and immunosuppressants show antiviral potential [22,29]. Although the mechanisms regarding the antiviral activities of azathioprine are not well-known, the proposed targets of antiviral activities via antirheumatic drugs and immunosuppressants are (1) attachment, (2) internalization (penetration), (3) uncoating, (4) biosynthesis, (5) assembly, and (6) egress seps [29]. Azathioprine, cyclosporine, hydroxyurea, minocycline, and mycophenolate also play a role in the biosynthesis step [22,29].

Mycophenolic acid inhibits dihydroorotate dehydrogenase, which is an essential enzyme for the synthesis of pyrimidine and IMPDH [29,30,31]. It was reported that mycophenolic acid has antiviral activities against human immunodeficiency virus and seasonal and avian influenza viruses [30,31]. Cyclosporin A inhibits T lymphocyte function by forming a complex with cyclophilin, and it targets cyclophilin D to inhibit permeability transition pore opening and rescue mitochondria from apoptosis [29,32,33]. It has been reported that cyclosporin A inhibits HCV replication [34,35]. In this study, we did not observe the inhibitory effects of mycophenolic acid and cyclosporin on HAV replication.

Previously, it was reported that a 21-year-old female with autoimmune hepatitis induced by prolonged HAV infection was successfully treated with 10 mg daily of prednisolone and 100 mg daily of azathioprine [36]. As breakthrough HAV infections, despite vaccination, are occasionally observed among immunocompromised populations [9], the development of anti-HAV drugs is crucial [17,37]. Although HAV infection is often self-convalesced in immunocompetent patients, it may show atypical manifestation, including acute liver failure, especially in patients undergoing transplant or immunosuppressive treatments [38,39,40]. With this study, we aimed to shed new light on the treatment of patients with HAV infection who are using immunosuppressive therapies.

One of the limitations of the present study is that we did not use animal models. In future research, it will be important to evaluate these molecules through in vivo analyses.

## 5. Conclusions

We conclude that azathioprine, an immunosuppressant, inhibits HAV replication without hepatocyte cytotoxicity. Azathioprine may be useful for the treatment of patients with HAV who are using immunosuppressants.

## Data Availability

The data are available in the article.

## Abbreviations

The following abbreviations are used in this manuscript:

HAV	Hepatitis A virus
IRES	Internal ribosomal entry site
SV40	Simian virus 40
RPMI	Rosen Park Memorial Institute
FBS	Fetal bovine serum
DMSO	Dimethyl sulfoxide
MOI	Multiplicity of infection
PBS	Phosphate-buffered saline
IC_50_	Half-maximal inhibitory concentration
MTS	Dimethylthiazol carboxymethoxyphenyl sulfophenyl tetrazolium
6-MP	6-Mercaptopurine
6-TUA	6-Thiouric acid
